# 5.D. Workshop: Challenges and opportunities for health systems in the era of multi-morbidity

**DOI:** 10.1093/eurpub/ckac129.289

**Published:** 2022-10-25

**Authors:** Organised by: Western University (Canada), Chair persons: Saverio Stranges (EUPHA-CHR), Arnaud Chiolero (Switzerland)

## Abstract

Multimorbidity, defined as the co-existence of two or more chronic conditions in the same individual, is affecting increasing segments of the population across both high-income and low-resource settings. The workshop will provide a comprehensive and global perspective on the clinical and public health burden of multimorbidity from a health system perspective, including presentations and speakers from Europe, Africa and North America. Current research and policy initiatives are still primarily focused on the clinical management of multimorbidity after it occurs as well as on the prevention of adverse events in those individuals who are already living with multimorbidity. There is still a significant need to develop and establish effective and equitable primary prevention strategies in order to avoid the occurrence of multimorbidity across populations. Also, health systems around the world will need to implement context-specific initiatives and changes to tackle the growing burden of multimorbidity in ageing populations, which will be constrained by available resources, societal and political priorities and values. Any potential health system transformations will need to reconcile the concomitant burden imposed by multimorbidity and other major public health priorities, including the current pandemic, the health impact of climate change and widening health disparities across marginalized populations.

**Key messages:**

• There is a significant need to develop effective and equitable primary prevention strategies in order to avoid the occurrence of multimorbidity.

• Health systems around the world will need to implement context-specific initiatives and changes, which will be constrained by available resources, societal and political priorities and values.

